# Acute symptomatic sinus bradycardia in a woman treated with pulse dose steroids for multiple sclerosis: a case report

**DOI:** 10.1186/s13256-015-0701-x

**Published:** 2015-09-24

**Authors:** Amartya Kundu, Timothy P. Fitzgibbons

**Affiliations:** 1 Department of Medicine, University of Massachusetts Medical School, 55 Lake Avenue North, Worcester, MA 01655 USA; 2 Department of Medicine, Cardiovascular Division, University of Massachusetts Medical School, 55 Lake Avenue North, Worcester, MA 01655 USA

**Keywords:** Sinus Bradycardia, Intravenous Methylprednisolone, Pulse Steroid Therapy, Multiple Sclerosis, Inappropriate sinus tachycardia.

## Abstract

**Introduction:**

Sinus bradycardia has been reported after administration of pulse dose steroids, although most cases have occurred in children and are asymptomatic. We report a case of acute symptomatic sinus bradycardia due to pulse dose steroids in a woman with multiple sclerosis. Interestingly, this patient also suffered from inappropriate sinus tachycardia due to autonomic involvement of multiple sclerosis.

**Case presentation:**

A 48-year-old Caucasian woman with multiple sclerosis and chronic palpitations due to inappropriate sinus tachycardia was prescribed a 5-day course of intravenous methylprednisolone for treatment of an acute flare. Immediately following the fourth dose of intravenous methylprednisolone, she developed dyspnea, chest heaviness, and lightheadedness. She was referred to the emergency department where an electrocardiogram showed marked sinus bradycardia (40 beats per minute). Initial laboratory test results, including a complete blood count, basic metabolic profile and cardiac biomarkers, were normal. She was admitted for observation on telemetry monitoring. Her heart rate gradually increased and her symptoms resolved. Her outpatient dose of atenolol, taken for symptomatic inappropriate sinus tachycardia, was resumed.

**Conclusions:**

Our patient’s acute symptoms were attributed to symptomatic sinus bradycardia due to pulse dose steroid treatment. Although several theories have been suggested to explain this phenomenon, the exact mechanism still remains unknown. It does not warrant any specific treatment, as it is a self-limiting side effect that resolves after discontinuing steroid infusion. Young patients who are free of any active cardiac conditions can safely be administered pulse dose steroids without monitoring. However, older patients with active cardiac conditions should have heart rate and blood pressure monitoring during infusion. Our patient also suffered from inappropriate sinus tachycardia, a manifestation of autonomic involvement of multiple sclerosis that has not been previously described. This case has implications for the pathogenesis and treatment of dysautonomia in patients with multiple sclerosis.

## Introduction

Multiple sclerosis (MS) is an autoimmune demyelinating disease of the central nervous system that frequently manifests with a waxing and waning course, characterized by lesions disseminated in both time and location. High-dose intravenous corticosteroid therapy, also known as pulse steroid therapy (PST), is commonly used to treat a wide range of autoimmune disorders because of its rapid anti-inflammatory effect and is considered to be standard therapy for treatment of acute flares of MS [1].

Although many side effects of intravenous steroid infusion are well established in the medical literature, PST is generally considered to have a good safety profile. Some of the commonly reported adverse effects following high-dose intravenous steroid infusion are hyperglycemia, gastrointestinal intolerance, and psychiatric manifestations such as euphoria or depression. Minor transient side effects include facial flushing, fluid retention, weight gain and parasthesias [1].

Sinus bradycardia is an uncommon adverse effect following steroid infusion and is very rarely symptomatic. Herein we report a case of symptomatic sinus bradycardia in a patient following PST with intravenous methylprednisolone given for treatment of an acute flare of MS.

## Case presentation

A 48-year-old Caucasian woman with a history of MS, smoking, and palpitations was prescribed a 5-day course of intravenous methylprednisolone (1 gram per day administered intravenously) as part of ongoing intensive therapy for a flare of MS. Her predominant symptoms were pain and numbness in her legs. One year prior, she had had a course of PST for similar symptoms. On this occasion, she noticed that her heart rate felt slow on days 2 and 3 following steroid infusions, but this was transient, and she felt normal soon afterward. Her documented heart rate during infusion on those days ranged from 81 to 96 beats per minute (bpm). However, during the infusion on day 4, she noticed that her chest started to feel heavy, she felt slightly lightheaded, and had difficulty breathing. She was transferred to the emergency department for evaluation of these symptoms. Prior to the infusion, she had only pain and numbness in her legs; the chest pain and dyspnea started after the infusion. On subsequent questioning, she denied any recent changes in her list of home medications. She had been taking atenolol for inappropriate sinus tachycardia (IST) for several years, and had taken her usual dose of 25mg that morning. Her usual resting heart ranged from 100 to 120bpm prior to starting atenolol, and 80 to 100bpm after starting atenolol.

**Fig. 1:**
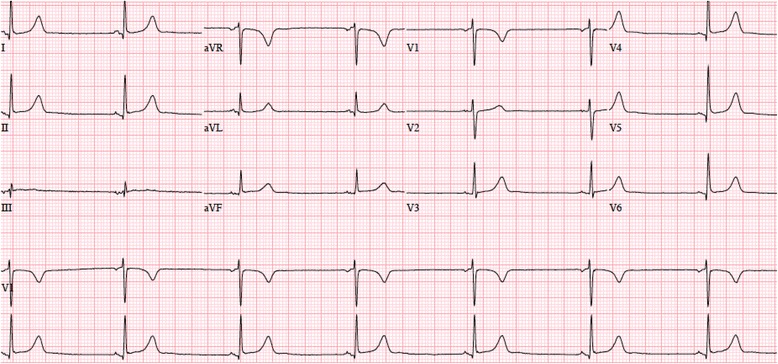
Electrocardiogram obtained upon admission showing sinus bradycardia at 42 beats per minute

**Fig. 2:**
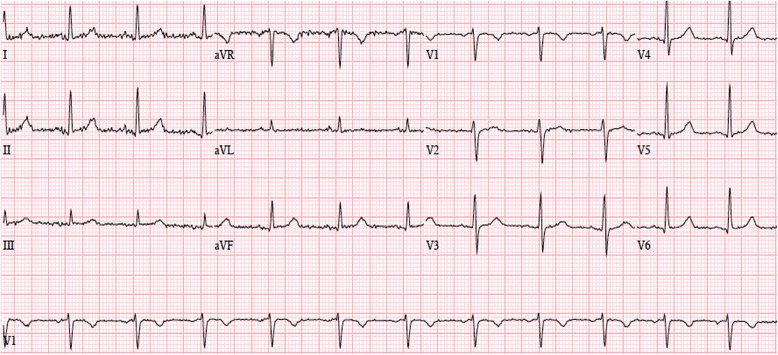
Electrocardiogram done 1-year prior demonstrating normal sinus rhythm at 78 beats per minute

On presentation to the emergency department, her blood pressure was 124/72mmHg, her pulse was 40bpm, and her oxygen saturation was 88% on room air. Results of the physical examination and laboratory tests were within normal limits. A 12-lead electrocardiogram (ECG) showed marked sinus bradycardia with a heart rate of 42bpm and normal PR (120msec), QRS (88msec) and QTc (402msec) intervals (Fig. 1). A prior ECG, done 1 year earlier, had shown normal sinus rhythm with a rate of 78bpm (Fig. 2). Cardiac biomarkers were normal and pulmonary embolism was excluded by a computed tomography (CT) pulmonary angiogram. She was admitted for observation on telemetry monitoring with supplemental oxygen therapy. With time, her oxygen saturation improved to normal on room air.

Telemetry monitoring showed persistence of sinus bradycardia for 8 hours without atrioventricular block or ventricular arrhythmias. Our patient denied any further chest tightness or lightheadedness. The next day, her heart rate increased to normal sinus rhythm at 80bpm. An echocardiogram was performed and showed normal biventricular size and function without valvular abnormalities. Her fifth (and final) dose of intravenous methylprednisolone was withheld. As our patient remained asymptomatic with a normal heart rate, she was discharged from the hospital soon after on her usual home dose of atenolol.

## Discussion

Although symptomatic sinus bradycardia following high-dose steroid infusion has been previously reported, it is an uncommon occurrence, and relatively underappreciated in the cardiovascular literature. Most of the prior reported cases have been asymptomatic, and all have recovered spontaneously over a variable period of time after stopping the steroid infusion [2–4].

Miura et al. reported sinus bradycardia occurring between 1 and 82% of children receiving intravenous methylprednisolone therapy for Kawasaki disease [5]. In a prospective study in adults by Tvede et al., five patients received high-dose intravenous methylprednisolone for treatment of rheumatoid arthritis [6]. All five patients experienced sinus bradycardia, although it was symptomatic in only one patient who reported chest tightness. It was self-limiting in all the patients, but it took as long as 7 days for the heart rate to return to normal.

Vasheghani-Farahani et al. conducted a study on 52 patients who were admitted to the hospital for PST for treatment of an acute flare of MS [7]. The purpose was to determine the effect of high-dose intravenous methylprednisolone on cardiac rhythms in patients with MS. All patients underwent continuous cardiac monitoring and a total of 167 sessions of PST were monitored. Patients with a history of cardiac disease and those receiving antiarrhythmic drugs or beta blockers were excluded from the study. The most common cardiac arrhythmia observed was sinus tachycardia, occurring in 83.8% of patients after steroid therapy. Sinus bradycardia was observed in 41.9% of the recorded rhythms after steroid infusion. Sinus bradycardia was found to be more common in patients who were male, smokers and those with autonomic disorders such as bladder and/or bowel sphincter disturbance [7].

Several hypotheses have been suggested to explain the pathophysiology of bradycardia following high-dose steroid infusion, but the exact mechanism still remains unknown. Both the rates of infusion, and the presence of underlying heart disease, are thought to increase the risk of occurrence [8]. Fujimoto et al. monitored ECG tracings, serum electrolyte levels as well as fractional excretion of sodium and potassium in 25 patients undergoing treatment with intravenous methylprednisolone for nephrotic syndrome [9]. Arrhythmias were observed in four of these patients. Serum potassium levels and fractional excretion of potassium significantly increased from baseline after PST. This suggests that abnormal cardiac rhythms may be caused due to sudden changes in potassium flux across the cell membrane [10, 11]. Another possible mechanism could be alterations in ionized calcium levels caused by formation of calcium phosphate complexes, which may be induced by the sodium phosphate contained in some commercially available preparations of methylprednisolone [12]. However our patient’s serum calcium and other electrolyte levels were normal on admission and prior to discharge.

Pudil et al. reported two patients who developed bradycardia following intravenous methylprednisolone where technetium-99m pyrophosphate myocardial scanning showed diffusely increased radionuclide accumulation in the myocardium that resolved on follow-up a few weeks later, suggesting that transient direct damage to the myocardium as a possible mechanism [13]. Our patient did not have a pharmacologic perfusion study, but echocardiography showed normal left ventricular function, making this explanation less likely. Although sinus bradycardia has most commonly been associated with high-dose PST, cases have also been reported following low-dose methylprednisolone infusion as well as oral prednisone therapy [14, 15]. Corticosteroids cause sodium retention and hypertension due to their intrinsic mineralocorticoid activity. Baroreceptor-mediated reflex bradycardia in response to hypertension caused by steroids is another potential explanation for bradycardia seen in patients treated with intravenous steroids [3]. Of note, our patient had no previous history of hypertension and her blood pressure level was normal throughout the course of observation. Finally, it has been suggested that bradycardia may simply be an idiosyncratic reaction to high-dose steroid infusion in a certain population of patients [16].

It is debatable whether routine cardiac monitoring is necessary for all patients receiving high-dose steroid therapy for treatment of MS, especially as a lot of PST sessions take place in the outpatient setting. Moreover, most cases of sinus bradycardia are self-limiting, asymptomatic, and do not require any treatment. White et al. recommended cardiac monitoring during PST for dermatologic patients who may have compromised skin integrity predisposing to electrolyte shifts, and in those with cardiac and renal disease [17]. It is probably prudent to proceed with a slow rate of steroid infusion and monitor select patients who have a history of cardiac disease or those who have experienced adverse effects following PST in the past. Interestingly, fingolimod, a recently approved sphingosine-1-phosphate inhibitor for use in relapsing-remitting MS, also causes sinus bradycardia and even second-degree atrioventricular block [18]. It is recommended that patients receive 6 hours of continuous ECG and blood pressure monitoring after the first dose of this oral disease-modifying agent [18].

Although involvement of the autonomic nervous system is common in MS, cardiovascular side effects are not. Autonomic symptoms usually include bowel, bladder, or sexual dysfunction. Postural orthostatic tachycardia syndrome (POTS) has been reported in MS, but IST has not. The latter can be difficult to diagnose as patients usually suffer from concomitant anxiety and/or pain [19]. Treatment is typically conservative, including beta blockers and anxiolytics, with electrophysiologic testing reserved for more severe cases [19]. Our patient has been managed effectively with the same conservative regimen.

We believe that this case highlights two unique features of MS that are not widely appreciated in the adult cardiovascular literature. One, pulse dose steroid treatment in patients with MS can cause acute asymptomatic and, more rarely, symptomatic sinus bradycardia. Newer disease-modifying agents, such as fingolimod, also cause bradyarrythmias, which can be more frequent and severe. Patient-specific factors such as active cardiac disease, smoking status or concomitant medications (that is, beta blockers) should be considered when administering these agents [7]. Second, autonomic involvement of MS can affect the cardiovascular system, and may cause POTS or IST. Care of patients with MS should be longitudinal and collaborative, involving primary care, neurology, cardiology and mental health professionals to consider all these factors.

## Conclusions

In conclusion, our patients’ symptoms of dyspnea, chest heaviness and lightheadedness were attributed to sinus bradycardia due to high-dose intravenous methylprednisolone therapy. The majority of prior reported cases have been asymptomatic and thus, symptomatic sinus bradycardia remains an extremely rare adverse effect of PST. It does not warrant any treatment as most cases are self-limiting and resolve after discontinuing steroid infusion. Cardiac monitoring is generally not needed if patients are young and free of active cardiac conditions, however patient-specific factors, such as the use of concomitant medications (that is, beta blockers) and smoking status should be considered when making this decision. Finally, chronic symptoms such as palpitations, syncope, or orthostatic intolerance in MS patients, should raise suspicion for cardiovascular effects of MS, which can include IST or POTS.

## Consent

Written informed consent was obtained from the patient for publication of this case report and any accompanying images. A copy of the written consent is available for review by the Editor-in-Chief of this journal.

## Abbreviations

bpm: beats per minute
CT: computed tomography
ECG: electrocardiogram
IST: inappropriate sinus tachycardia
MS: multiple sclerosis
PST: pulse steroid therapy
POTS: postural orthostatic tachycardia syndrome
